# Regulation and function of the mammalian tricarboxylic acid cycle

**DOI:** 10.1016/j.jbc.2022.102838

**Published:** 2022-12-26

**Authors:** Paige K. Arnold, Lydia W.S. Finley

**Affiliations:** 1 Cell Biology Program, Sloan Kettering Institute, Memorial Sloan Kettering Cancer Center, New York, New York, USA; 2 Louis V. Gerstner Jr. Graduate School of Biomedical Sciences, Memorial Sloan Kettering Cancer Center, New York, New York, USA

**Keywords:** tricarboxylic acid cycle, cell metabolism, bioenergetics, Krebs cycle, citric acid cycle, ACL, ATP-citrate lyase, ACO, aconitase, ACSS, acetyl-CoA synthetase, αKG, alpha-ketoglutarate, BCAT1, branched-chain amino acid transaminase 1, CCL, citryl-CoA lyase, CPT1, carnitine acetyltransferase I, CS, citrate synthase, d-2HG, d-2-hydroxyglutarate, ESC, embryonic stem cell, ETC, electron transport chain, FH, fumarate hydratase, GDH, glutamate dehydrogenase, GPD, glycerol 3-phosphate dehydrogenase, GOT2, glutamic-oxaloacetic transaminase 2, HFSC, hair follicle stem cell, HIF, hypoxia-inducible transcription factor, IDH, isocitrate dehydrogenase, IRE, iron-responsive element, ISC, intestinal stem cell, LDH, lactate dehydrogenase, LDHA, lactate dehydrogenase A, MDH1, malate dehydrogenase 1, MDH2, malate dehydrogenase 2, MPC, mitochondrial pyruvate carrier, OAA, oxaloacetate, OGDH, oxoglutarate dehydrogenase, OXPHOS, oxidative phosphorylation, PC, pyruvate carboxylase, PDHC, pyruvate dehydrogenase complex, PDK, pyruvate dehydrogenase kinase, PDP, pyruvate dehydrogenase phosphatase, ROS, reactive oxygen species, rTCA, reductive TCA, SDH, succinate dehydrogenase, TCA, tricarboxylic acid, TET, ten–eleven translocation

## Abstract

The tricarboxylic acid (TCA) cycle, otherwise known as the Krebs cycle, is a central metabolic pathway that performs the essential function of oxidizing nutrients to support cellular bioenergetics. More recently, it has become evident that TCA cycle behavior is dynamic, and products of the TCA cycle can be co-opted in cancer and other pathologic states. In this review, we revisit the TCA cycle, including its potential origins and the history of its discovery. We provide a detailed accounting of the requirements for sustained TCA cycle function and the critical regulatory nodes that can stimulate or constrain TCA cycle activity. We also discuss recent advances in our understanding of the flexibility of TCA cycle wiring and the increasingly appreciated heterogeneity in TCA cycle activity exhibited by mammalian cells. Deeper insight into how the TCA cycle can be differentially regulated and, consequently, configured in different contexts will shed light on how this pathway is primed to meet the requirements of distinct mammalian cell states.

Cellular metabolism comprises a complex network of biochemical reactions that convert nutrients into metabolic building blocks that fuel the growth and survival of living organisms. Metabolic processes require energy exchange, which is achieved through oxidation–reduction reactions that transfer high-energy electrons from one molecule onto another. Outputs of metabolic pathways can broadly be separated into three categories: energy, reducing equivalents, and macromolecular precursors. These outputs are required in all living cells, but the relative degree to which cells rely on each output is dependent upon the specific requirements of distinct cell types and cell states. Accordingly, myriad signaling pathways and regulatory networks control the balance between catabolic pathways, which break down molecules to harness chemical energy, and anabolic pathways, that orchestrate macromolecular synthesis.

At the nexus of both catabolic and anabolic metabolism lies the tricarboxylic acid (TCA) cycle, a broadly conserved metabolic pathway consisting of a cyclic series of chemical reactions that harness high-energy electrons from fuel sources (1, 2, 3). The chemical reaction that initiates each “turn” of the TCA cycle is the condensation of the four-carbon metabolite oxaloacetate (OAA) with the two-carbon molecule acetyl-CoA to generate citrate. Subsequent reactions oxidize citrate to produce two molecules of CO_2_ and one GTP or ATP molecule (Fig. 1) (3). During each turn of the cycle, three hydride ions (six electrons) are transferred to three NAD^+^ molecules and one pair of hydrogen atoms (two electrons) are transferred to one FAD molecule, producing four reducing equivalents total (three NADH and one FADH_2_) (Fig. 1). Importantly, each turn of the cycle ends with the regeneration of the starting molecule, OAA. Thus, OAA acts as a catalyst in the TCA cycle: only a small amount is required for the oxidation of large amounts of acetyl-CoA (4).

**Figure 1:**
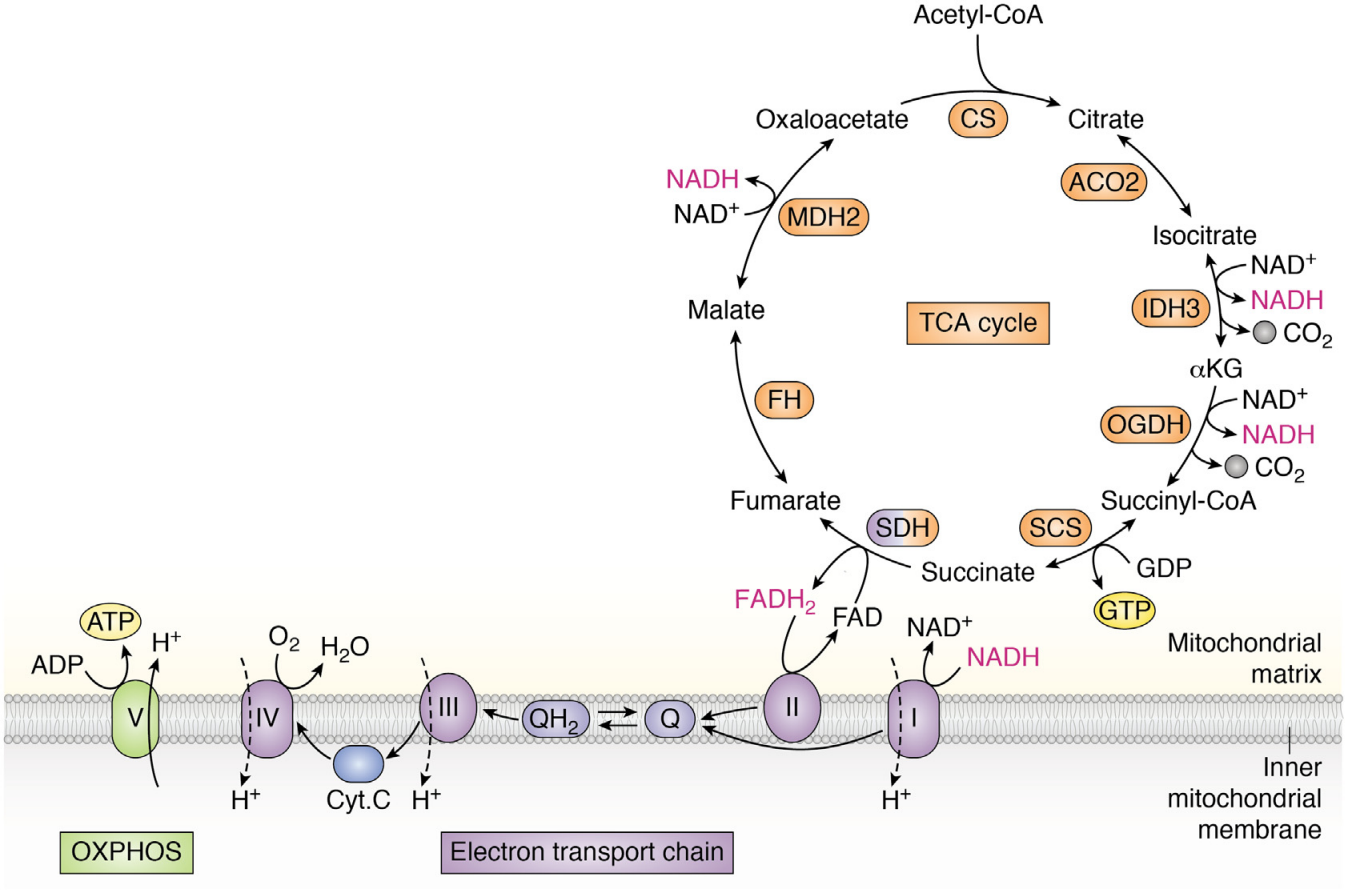
**Overview of the tricarboxylic acid (TCA) cycle and electron transport chain (ETC).** The TCA cycle starts when the two-carbon molecule acetyl-CoA combines with four-carbon oxaloacetate to form citrate, a reaction catalyzed by citrate synthase (CS). Citrate is then converted to isocitrate by aconitase 2 (ACO2). Isocitrate is decarboxylated to alpha-ketoglutarate (αKG) in an NAD^+^-dependent manner by isocitrate dehydrogenase 3 (IDH3) or in an NADP+-dependent manner by isocitrate dehydrogenase 2 (IDH2), releasing carbon dioxide (CO_2_). αKG undergoes decarboxylation to succinyl-CoA *via* the oxoglutarate dehydrogenase complex (OGDH), producing NADH and releasing CO_2_. Succinyl-CoA is then converted to succinate by succinyl-CoA synthetase (SCS). This is the only substrate-level phosphorylation step in the TCA cycle, as it is coupled to the generation of GTP or ATP. Succinate is converted to fumarate by succinate dehydrogenase (SDH) complex, a multisubunit enzyme complex that participates in both the TCA cycle and the electron transport chain (ETC). SDH reduces FAD to FADH_2,_ which donates its electrons to complex II. Fumarate is converted to malate by fumarate hydratase (FH). Malate dehydrogenase 2 (MDH2) converts malate to oxaloacetate in an NAD+-dependent manner, regenerating the starting molecule and supporting the next turn of the cycle. Note: most TCA cycle reactions are reversible. Substrate oxidation reactions are coupled to reduction of electron carriers NAD^+^ and FAD: each complete turn of the TCA cycle generates three NADH and one FADH_2_ molecules, which donate their electrons to complex I and complex II, respectively. These reducing equivalents are reoxidized upon donating their electrons to the ETC, supporting continued TCA cycle activity. Electrons donated to complexes I and II are transferred to ubiquinone (Q), reducing it to ubiquinol (QH_2_). Ubiquinol is reoxidized to ubiquinone upon passing its electrons to complex III, which transfers electrons to cytochrome C (Cyt C). Cyt C passes its electrons onto complex IV, which then transfers its electrons to the terminal electron acceptor, oxygen (O_2_), forming water (H_2_O). As electrons are transferred through the ETC and eventually onto oxygen, complexes I, III, and IV pump protons across the inner mitochondrial membrane. This proton pumping establishes a proton gradient that is used by complex V, or ATP synthase, to generate ATP from ADP, a process known as oxidative phosphorylation (OXPHOS). TCA cycle enzymes are colored in *orange*; SDH is colored *blue* and *orange* because it participates in both the TCA cycle and the ETC. Reducing equivalents are shown in *pink*.

The TCA cycle itself neither consumes molecular oxygen nor produces meaningful amounts of ATP; rather, the TCA cycle removes electrons (reducing equivalents) from inputs (*e.g.*, acetyl-CoA) and transfers them to electron carriers that deposit their electrons onto the electron transport chain (ETC). Electron funneling through the complexes of the ETC is coupled to generation of the mitochondrial membrane potential that is ultimately used to power the production of the cellular currency (ATP) in a process known as oxidative phosphorylation (OXPHOS), as oxygen serves as the terminal acceptor for electrons that transit through the ETC (Fig. 1) (3). The TCA cycle is thus linked to oxygen consumption, as the oxidized electron carriers required to continue to turn the cycle are regenerated by the ETC, and the concerted activity of these two pathways allows for the generation of significant amounts of ATP. In this configuration, the central function of the TCA cycle is to convert fuel sources into energy, and therefore, the TCA cycle is generally considered a catabolic process. However, several chemical intermediates of the TCA cycle also serve as critical precursors for biosynthetic reactions, which we will discuss in detail later (5, 6). Because the TCA cycle functions in both catabolic and anabolic capacities, it is considered an amphibolic pathway (7, 8).

Cells must carefully calibrate anabolic and catabolic pathways to ensure balance between nutrient supply and demand. Given the central role of the TCA cycle in both provisioning key anabolic substrates and in maintaining energy production, it is not surprising that TCA cycle activity is under tight physiologic regulation. Multiple metabolic signals control both TCA cycle inputs and directionality, and increasing work demonstrates the importance of such TCA cycle flexibility for optimizing cellular fitness both under physiological conditions and in the context of disease. In mammals, the consequences of disrupting TCA cycle function can be found in a group of disorders known as inborn errors of metabolism, which are caused by inherited mutations in genes encoding metabolic enzymes. Human patients who present with mutations in TCA cycle–associated genes display neonatal symptoms, developmental defects, and failure to thrive (9, 10). These observations support the notion that the TCA cycle plays a significant role in sustaining mammalian tissue function. The goal of this review is to revisit the wiring of the TCA cycle in detail, leveraging both historical work and more recent studies to provide an updated view of the mammalian TCA cycle as a dynamic metabolic network at the heart of cell biology.

## Evolutionary perspectives on the TCA cycle

Cellular life emerged approximately four billion years ago, at a time when earth’s atmosphere is predicted to have been largely devoid of oxygen (11). The existence of life in any capacity on earth requires the generation of basic cellular constituents that enable organization necessary to combat entropy. Ancient metabolic pathways thus must have acted to assemble organic molecules from inorganic precursors for the synthesis of biomolecules, like proteins, lipids, and nucleic acids. A major theory in the study of primordial metabolism is that these ancient anabolic pathways originated spontaneously based on geochemical conditions before the emergence of enzymes, genetic material, or cells (12). The TCA cycle plays a central role in theories for the chemical origins of life because it supplies acetyl-CoA, pyruvate, OAA, succinate, and alpha-ketoglutarate (αKG), the five universal metabolic precursors for biosynthetic molecules (*e.g.*, lipids, glucose, nucleic acids, amino acids, and cofactors). Suggestively, many of the reactions of the oxidative TCA cycle can occur nonenzymatically in the presence of iron as a catalyst and oxidizing agents other than oxygen (13, 14, 15). However, given that prebiotic chemistry would necessarily have initially required the production, not the breakdown, of organic molecules, primordial metabolism likely centered on reductive CO_2_-fixing pathways.

**Figure 2:**
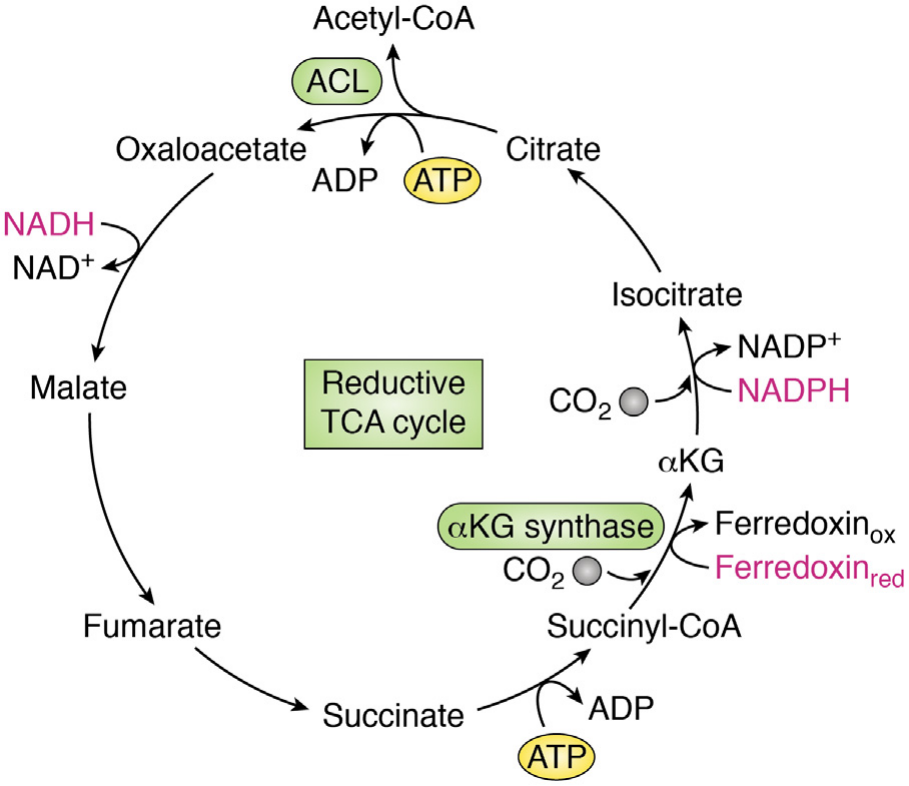
**The reductive tricarboxylic acid (TCA) cycle.** A simplified schematic depicting the reductive TCA cycle or reverse Krebs cycle. Most reactions of this cycle are the same as those of the oxidative TCA cycle but in reverse and are catalyzed by similar enzymes. The major exceptions include (1) cleavage of citrate to form oxaloacetate and acetyl-CoA and (2) the production of alpha-ketoglutarate (αKG) from succinyl-CoA. Citrate cleavage requires ATP and is carried out by ATP-citrate lyase (ACL) or the related citryl-CoA lyase and citryl-CoA synthase enzymes. Conversion of succinyl-CoA to αKG, mediated by αKG synthase, is highly energetically unfavorable and thus requires a strong reducing agent in the form of reduced ferredoxin (Ferredoxin_red_). While the oxidative TCA cycle combusts carbon and produces reducing equivalents that drive ATP synthesis, the reductive TCA cycle consumes ATP and reducing equivalents to assimilate carbon and produce acetyl-CoA. Reducing equivalents are shown in *pink*.

Clues about primordial metabolism have been sought by studying the metabolism of CO_2_-fixing chemoautotrophs, which lie at the root of the tree of life, and thus may offer insights into the earliest biosynthetic pathways (12). A CO_2_-fixing pathway of particular interest found in eubacteria and archaea and both aerobes and anaerobes is the reductive TCA cycle (rTCA cycle) or reverse Krebs cycle (16). The rTCA cycle consists of a cyclic series of chemical reactions that are essentially those of the TCA cycle but in reverse (Fig. 2) (17). Accordingly, while the oxidative TCA cycle oxidizes acetyl-CoA to CO_2_ to generate reducing equivalents and ATP, the rTCA cycle captures CO_2_ to generate acetyl-CoA in a process requiring electron donors and ATP. The rTCA cycle is an appealing candidate for early prebiotic metabolism because, similar to the oxidative TCA cycle, it produces the five universal precursors to biological metabolism (12, 14, 18). Intriguingly, certain reaction sequences of the cycle have been shown to occur nonenzymatically in the presence of minerals under UV radiation or using metal ions under acidic and aqueous conditions, not unlike those proposed on early Earth (12, 14, 19, 20). However, demonstration of the C-C bond forming reactions of the rTCA cycle has yet to be achieved efficiently and nonenzymatically under experimental conditions, raising the possibility that this portion of the pathway required organic catalysts and, thus, emerged later (21, 22).

Ultimately, the ability of the rTCA cycle and/or other anabolic pathways to produce organic molecules allowed for the emergence of enzymes that facilitated these biochemical reactions. Many of the enzymes of the oxidative and forward TCA cycle are reversible and, thus, function similarly to the enzymes that comprise the rTCA cycle. One of the biggest differences between the two cycles is that the rTCA cycle requires the conversion of succinyl-CoA to αKG. This reaction, mediated by an αKG synthase, is highly unfavorable and requires a strong reducing agent in the form of reduced ferredoxin (18). In the forward TCA cycle, citrate synthase (CS) is the key enzyme that condenses acetyl-CoA with OAA to form citrate. The rTCA cycle, on the other hand, requires that citrate be cleaved to liberate acetyl-CoA and regenerate OAA, a reaction that is catalyzed by the ATP-citrate lyase (ACL) enzyme. Human ACL contains a citryl-CoA synthetase module and a citryl-CoA lyase (CCL) domain, which coordinate to catalyze the multistep cleavage of citrate (23). However, in the rTCA cycle active in the deep branching bacterial phylum Aquificae, CCL and citryl-CoA synthetase function as two distinct enzymes that mediate citrate cleavage in a stepwise manner. Recently, cross-kingdom structural analysis revealed conserved structural features between CCL in Aquificae and CS in a species of Archaea, suggesting that the process of citrate condensation originated from citrate cleavage during evolution (23). Moreover, the CS reaction, which was previously considered irreversible, can proceed in the reverse direction in certain anaerobes, and this reversibility is favored under high CO_2_ conditions (24, 25, 26). These findings strengthen the notion that the rTCA cycle may represent both a vestige of prebiotic metabolism and an evolutionary precursor for the oxidative TCA cycle (18, 27).

The oxygenation of Earth’s atmosphere over time increased the availability of molecular oxygen, whose function as an electron acceptor facilitates oxidation of organic molecules derived from carbon assimilation (21, 28). This development likely enabled the emergence of the oxidative TCA cycle and its coupling with OXPHOS, allowing the transfer of electrons derived from the breakdown of carbon substrates onto molecular oxygen. The evolution of increasingly complex TCA cycle enzymes was a critical development allowing for both improved efficiency of this metabolic pathway and enhanced capacity for regulation of its activity (14). Being able to fine-tune the function of metabolic pathways like the TCA cycle—in particular, to toggle between catabolic and anabolic activity—set the stage for early unicellular life forms to evolve into multicellular organisms, which require coordination of metabolic activity beyond that dictated by local nutrient availability (29, 30).

## Discovery of the TCA cycle

At the beginning of the 20th century, cellular respiration remained one of the major unsolved problems in biology. Scientists knew that cellular respiration involved oxygen consumption and also suspected that the process was somehow linked to the catabolism of nutrients like carbohydrates. It was also known that dicarboxylic acids played some sort of role in respiration, but it was assumed that these compounds acted as substrates and thus were consumed in the process. Moreover, the intermediate reaction sequences and fundamental biochemistry of respiration had yet to be worked out. Seeking to understand the oxidative breakdown of carbohydrates, the Hungarian scientist Albert Szent-Györgyi turned to pigeon breast muscle as an experimental system because of its high rates of respiration and ease of accessibility. In 1935, Szent-Györgyi worked out the sequence of reactions from succinate to fumarate to malate to OAA and demonstrated the rapid oxidation of these substances by pigeon muscle suspensions (31, 32). From this work, Szent-Györgyi hypothesized that dicarboxylic acids catalytically promoted oxygen uptake (32). Support for this idea came the following year when Stare (33) and Baumann found that very small quantities of dicarboxylic acids were sufficient to cause an increase in oxygen consumption and that this increase was beyond what was necessary for the oxidation of the quantities of substrate added. Significantly, they also found that added dicarboxylic acids were not used up and could still be detected in the medium, indicating that these compounds were functioning in a catalytic capacity (33). In 1937, Hans Krebs (1, 32) showed that succinate could be synthesized by animal tissues in the presence of pyruvate, leading him to speculate that succinate may arise from citrate oxidation downstream of pyruvate. A key finding in that same year came from Martius and Knoop (34), who found that αKG is a product of citrate oxidation and worked out the sequence of reactions from citrate to succinate.

These crucial experiments teed up the findings of Hans Krebs and his colleague William Johnson (2) in *Enzymologia* in 1937, in which they discovered that citrate was readily oxidized by minced pigeon breast muscle and that citrate addition increased oxygen uptake beyond the amount necessary for the complete oxidation of the added citrate, indicating that citrate itself was stimulating consumption of molecular oxygen. Moreover, they found that citrate did not disappear during this process, indicating that it was being continuously reformed (2, 32). Their work also showed that large quantities of citrate could be synthesized anaerobically by muscle in the presence of OAA and that OAA, when added to muscle, condensed with two carbon atoms from an unknown precursor to form citrate (2). By adding malonate, an inhibitor of succinate oxidation, they found that succinate accumulated when OAA was added to muscle, implying that this series of reactions was cyclical in nature (2, 32). In subsequent work, Krebs (4) determined that the two carbon atoms that condensed with OAA to form citrate were derived from pyruvate and that oxygen consumption in muscle increases when pyruvate is added. Later work by Lipmann *et al.* (35, 36) elucidated that the two-carbon molecule required for citrate synthesis was, in fact, acetyl coenzyme A or acetyl-CoA.

## Wiring of the TCA cycle

**Figure 3:**
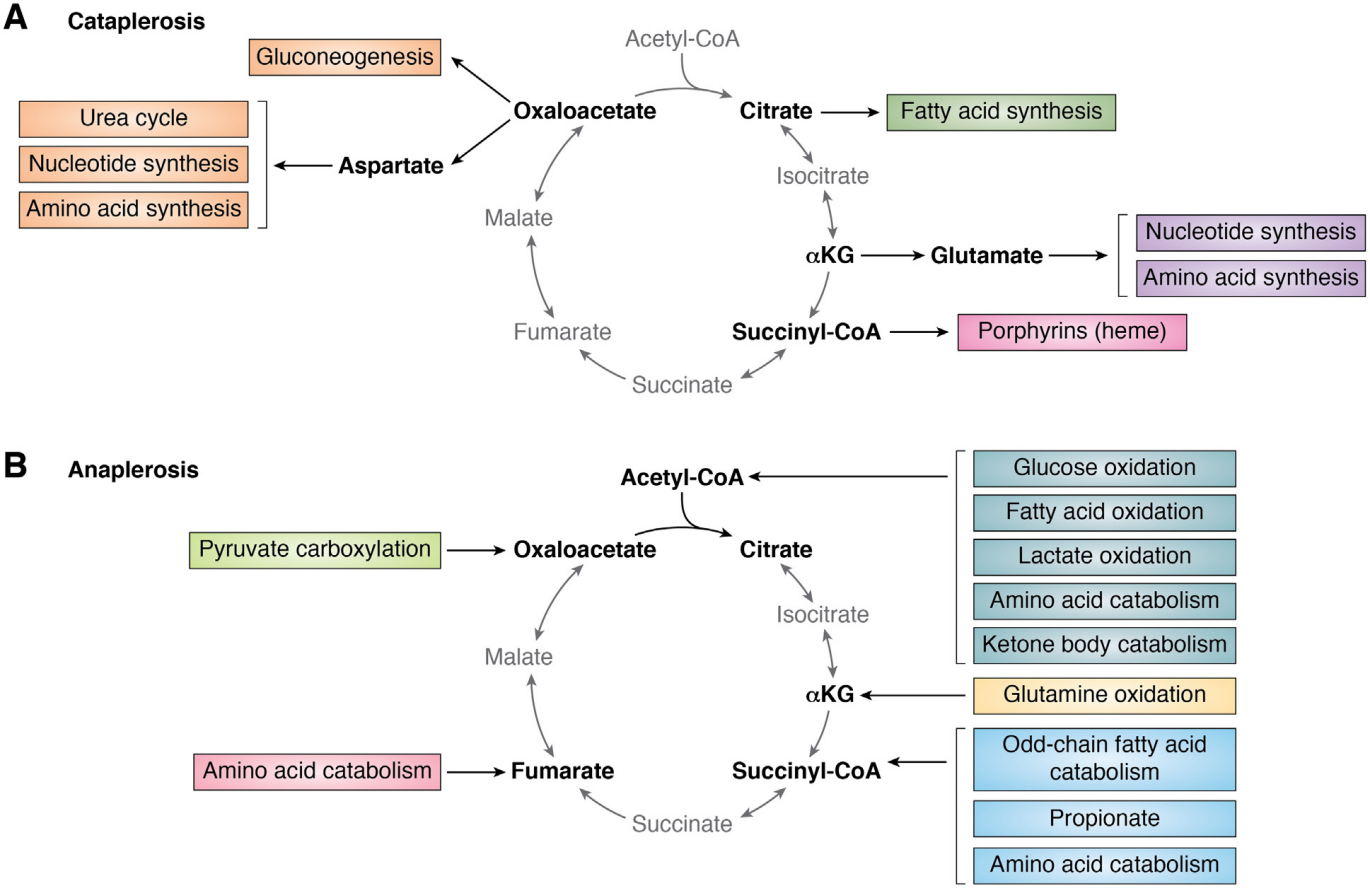
**Outputs and inputs into the tricarboxylic acid (TCA) cycle.** A functioning TCA cycle requires a continuous pool of acetyl-CoA and supply of TCA cycle intermediates that can be used to synthesize oxaloacetate. *A*, removal of TCA cycle intermediates (“cataplerosis”) occurs at multiple steps of the cycle to supply precursors for biosynthetic processes or feed into other metabolic pathways. *B*, replacement of TCA cycle intermediates (“anaplerosis”) is required to support continuous production of oxaloacetate. Sources of anaplerosis are shown in this panel. Several pathways either produce acetyl-CoA directly or produce pyruvate, an indirect source of acetyl-CoA through the activity of the pyruvate dehydrogenase complex.

In theory, OAA regeneration allows for infinite turning of the TCA cycle, assuming a continuous supply of acetyl-CoA. Following multiple turns of the cycle, the metabolic intermediates of the TCA cycle can be entirely derived from and replaced by acetyl-CoA carbons (5). However, TCA cycle intermediates can be siphoned from the cycle to feed into other metabolic pathways or to supply precursors for macromolecule biosynthesis, a process termed “cataplerosis” (37). For example, mitochondrial citrate can be exported to the cytoplasm and metabolized by ACL to liberate acetyl-CoA, which is required for *de novo* lipid synthesis and protein acetylation (38, 39, 40). The metabolite αKG can be converted to glutamate, which in turn is diverted from the cycle and used in the synthesis of amino acids and nucleotides. Succinyl-CoA may be siphoned from the cycle to serve as a precursor of porphyrins like heme (41). OAA itself provides the carbon backbone for the amino acid aspartate, a critical input into the urea cycle and protein and nucleotide biosynthesis, and may be converted to phosphoenolpyruvate, a substrate for gluconeogenesis (41, 42, 43). Cataplerosis of any TCA cycle intermediate requires compensatory input to sustain TCA cycle activity, a process termed “anaplerosis” (37). Thus, in living cells, robust and sustained TCA cycle function requires both a continuous source of acetyl-CoA and replenishment of TCA cycle intermediates (particularly OAA) *via* anaplerotic reactions (Fig. 3). Here, we outline the various sources of acetyl-CoA production and OAA regeneration.

### Sources of acetyl-CoA

The TCA cycle is the final step for oxidation of all cellular nutrients, including glucose, fatty acids, and amino acids, which primarily enter the cycle at the level of acetyl-CoA. While the mechanisms dictating a cell’s preferred source of acetyl-CoA are not well understood, recent work has found that different tissues in the body have distinct fuel preferences (44), raising the possibility that the source of acetyl-CoA generation is dependent on cell type and cell state. The diverse routes by which nutrients are converted to acetyl-CoA are described later.

#### Glucose oxidation

Pyruvate functions as a significant source of acetyl-CoA for the TCA cycle in most mammalian cells (45). In cultured cells, pyruvate is largely produced from glucose *via* glycolysis; *in vivo*, circulating lactate provides an additional major source of tissue pyruvate (46, 47, 48). Pyruvate is imported into the mitochondrial matrix by the mitochondrial pyruvate carrier (MPC) (49, 50, 51). Once in the mitochondrial matrix, pyruvate can undergo oxidative decarboxylation to form acetyl-CoA. This irreversible reaction is catalyzed by the pyruvate dehydrogenase complex (PDHC), a supramolecular assembly of multiple catalytic subunits (41). The PDHC is allosterically inhibited by NADH, acetyl-CoA, and ATP, making it a potent sensor of TCA cycle activity and thus a critical node of TCA cycle regulation (discussed further) (52). By converting the glycolytic product pyruvate into a substrate for TCA cycle oxidation, the PDHC functions as a gatekeeping enzyme that links glycolysis to the TCA cycle and mitochondrial respiration.

Glycolysis-derived pyruvate has alternative fates outside the mitochondrial oxidation. Pyruvate can be used as a gluconeogenic substrate, but acetyl-CoA cannot; consequently, the PDHC reaction is a key step that is tightly regulated to control whether pyruvate is used in the TCA cycle or for gluconeogenesis (53, 54). Beyond its critical role in gluconeogenesis, pyruvate utilization also has implications for redox homeostasis. Lactate dehydrogenase (LDH) can reduce pyruvate to lactate in the cytosol, and this reaction is coupled with oxidation of NADH to NAD^+^. In the absence of oxygen, this process of fermenting pyruvate to form lactate, also known as anaerobic glycolysis, occurs in mammals as a means of sustaining biosynthetic processes under hypoxia (41). However, in 1924, Otto Warburg (55, 56) found that cancer cells produce significant amounts of lactate even in the presence of oxygen. Subsequent investigation has revealed that proliferating cells significantly engage in this process, termed aerobic glycolysis or the Warburg effect. While the field continues to debate the underlying benefits of engaging in aerobic glycolysis, one clear advantage to the process is that it allows cells to robustly regenerate NAD^+^ in the cytosol (57). Cytosolic NAD^+^ is used during the conversion of glyceraldehyde-3-phosphate to 1,3-bisphosphoglycerate and, thus, is required to sustain continuous flux through glycolysis (6). Glycolysis provides carbon that supports nucleotide and lipid biosynthesis, and some glycolytic intermediates are precursors for amino acid biosynthesis (6). Therefore, glycolysis provides several biosynthetic advantages to proliferating cells beyond producing pyruvate. Proliferating cells thus must balance use of pyruvate toward oxidation in the TCA cycle with demand for cytosolic NAD^+^ regeneration to support continued glycolytic flux.

#### Lactate oxidation

While glucose-derived pyruvate is a considerable source of acetyl-CoA carbons for TCA cycle oxidation in cultured cells *in vitro*, recent work has revealed that glucose may not be the preferred pyruvate source of cells *in vivo* (46, 47, 48). In rapidly growing cells, the LDH-mediated conversion of pyruvate to lactate is a major source of cytosolic NAD^+^ that supports sustained glycolytic flux. Proliferating cells in culture typically excrete this lactate as a waste product to support redox and pH homeostasis (46). However, recent studies *in vivo* have demonstrated that cells import lactate from the circulation *via* monocarboxylate transporter 1 and subsequently oxidize lactate to form pyruvate, resulting in a large fraction of pyruvate and downstream TCA cycle intermediates being derived from circulating lactate (46, 47). In most tissues and some tumors, the contribution of circulating lactate to TCA cycle intermediates exceeds that of glucose, supporting the idea that lactate is a fundamental TCA cycle substrate in living organisms (46, 47). As glucose uptake and usage are under growth factor control (58, 59, 60), this observation raises the possibility that a basal level of TCA cycle oxidation in cells can persist independent of exogenous growth factor stimulation. Future work should be aimed at understanding the compartmentalization of lactate oxidation, as the conversion of lactate to pyruvate is coupled to reduction of NAD^+^ to NADH. Absent compensatory measures, this change in the cytosolic NAD^+^/NADH ratio will hamper metabolic pathways—like glycolysis—that depend on oxidized NAD^+^. Suggestively, one study found that LDH can localize to the mitochondria, raising the possibility that lactate can be directly imported into the mitochondria for further oxidation (61). Mitochondrial import and catabolism of lactate would prevent the buildup of cytosolic NADH while allowing for the mitochondrial capture of both carbon and reducing equivalents (61).

#### Fatty acid oxidation

Acetyl-CoA is both the precursor for fatty acid synthesis and the final product of fatty acid breakdown for oxidation in the TCA cycle. To separate these functions, cells synthesize fatty acids in the cytosol *via* ACL-mediated citrate cleavage and, conversely, import fatty acids into the mitochondrial matrix for degradation and subsequent oxidation. The process of breaking down fatty acids to produce acetyl-CoA, known as β-oxidation, supplies the majority of acetyl-CoA for TCA cycle oxidation in certain tissues such as the heart (62). Because the mitochondrial membrane is impermeable to acyl-CoAs, fatty acids must first be transported from the cytosol into the mitochondrial matrix *via* the carnitine shuttle prior to undergoing β-oxidation (41). Fatty acids are first converted to fatty acyl-carnitines on the outer mitochondrial membrane by carnitine acetyltransferase I (CPT1), then transported across the membrane *via* the carnitine-translocase protein, and reconverted back to acyl-CoA esters inside the mitochondrial matrix by carnitine acetyltransferase II (41). Mitochondrial fatty acyl-CoAs then undergo β-oxidation in a sequential degradation reaction coordinated by four enzymes (63). The four steps of the β-oxidation process are repeated until the fatty acyl-CoA is entirely oxidized, with each iteration shortening the molecule by two carboxy-terminal carbons, which are liberated as acetyl-CoA (63). To prevent the futile cycle of simultaneous fatty acid synthesis and oxidation, the rate-limiting enzyme of fatty acid oxidation—CPT1—is regulated in both a transcriptional and an allosteric manner. Sensing of free fatty acid availability within the cell primarily occurs through peroxisome proliferator–activated receptors, which function as fatty acid–activated transcription factors that drive the expression of CPT1 (64, 65). CPT1 itself is allosterically inhibited by the fatty acid synthesis intermediate malonyl-CoA (41, 63).

#### Amino acid, ketone body, and acetate catabolism

Acetyl-CoA can also be derived from the breakdown of amino acids and ketone bodies. Removal of the amino group from ketogenic amino acids, including lysine and the branched-chain amino acids, leucine and isoleucine, results in a carbon skeleton that can be catabolized to acetyl-CoA directly or the ketone body acetoacetate (66, 67). Acetoacetate can be converted to acetyl-CoA by first undergoing conversion to acetoacetyl-CoA by β-ketoacyl-CoA transferase followed by cleavage by thiolase (41). In differentiated adipocytes, catabolism of branched-chain amino acids accounts for almost a third of cellular acetyl-CoA pools (68). Amino acids can also contribute to acetyl-CoA pools *via* a pyruvate intermediate. For example, catabolism of glucogenic amino acids, including serine and cysteine, and the transamination of alanine by alanine aminotransferase produces pyruvate, which can be converted to acetyl-CoA by PDHC. Beyond amino acids, the ketone body β-hydroxybutyrate can also be converted to acetoacetate, providing another potential source of acetyl-CoA.

Acetate can function as a source of acetyl-CoA through the activity of acetyl-CoA synthetase (ACSS), which catalyzes the ATP-dependent ligation of acetate and CoA to produce acetyl-CoA. In cancer cells, citrate becomes labeled following supplementation of cells with isotopically labeled acetate ([^13^C]acetate), indicating that acetate can undergo oxidation in the TCA cycle in cultured cell lines (69). The relevance of acetate as a potential fuel source has also been observed *in vivo*: in glioblastoma tumors resected from patients, up to half of the intramitochondrial acetyl-CoA pool is derived from circulating acetate (70). Distinct isoforms of ACSS localize specifically to the cytosol and mitochondria, but how conversion of acetate to acetyl-CoA is coordinated between these two compartments remains poorly understood (71).

### TCA cycle anaplerosis

#### Glutamine oxidation

The preferred anaplerotic substrate in most proliferating cells growing in culture is glutamine, the most abundant circulating amino acid in mammals (30, 72, 73, 74). While glutamine can be synthesized directly by most mammalian cells, it has been recognized for several decades that glutamine supplementation is necessary for the growth and viability of many cultured cell lines, particularly cancer cell lines (72, 75, 76). Glutamine taken up by cells through transporters such as alanine–serine–cysteine transporter 2 and l-type amino acid transporter 1 is converted to glutamate by glutaminase, producing ammonia, or through nitrogen-donating reactions involved in purine and pyrimidine nucleotide synthesis (77). Glutamate can then be converted to αKG, which can enter the TCA cycle for further oxidation.

Production of αKG from glutamate occurs through two mechanisms: (1) by deamination *via* glutamate dehydrogenase (GDH), releasing ammonia and the reducing equivalent NAD(P)H or (2) by transaminases that transfer the amino group from glutamate to a keto-acid, generating αKG and an amino acid (78). Notably, transamination is freely reversible, meaning that aspartate aminotransferases (glutamic-oxaloacetic transaminase 1; glutamic-oxaloacetic transaminase 2 [GOT2]) can alternatively directly generate glutamate and OAA using αKG and aspartate. Cells can become particularly reliant on glutamine oxidation *via* GDH when their ability to oxidize glucose is impaired, for example, upon MPC inhibition (79). However, under normal and glucose-replete conditions, transamination is the dominant reaction that drives glutamine anaplerosis in cultured cells (79, 80, 81, 82). The preferential reliance on GOT2 for mitochondrial anaplerosis may be a byproduct of tissue-specific expression patterns and considerable allosteric regulation of GDH (83). Notably, GDH is potently inhibited by NADH, GTP, and ATP (84), which may be abundant in mitochondria of cultured cells. The importance of this allosteric regulation is underscored by patients harboring germline mutations in the GTP-binding region of GDH: persistently high GDH activity drives hyperinsulinism–hyperammonemia syndrome, marked by aberrant glutamate catabolism and mitochondrial ATP production leading to excessive insulin release from pancreatic beta cells (83, 85). GOT2-driven glutamate metabolism thus offers several benefits to proliferating cells: GOT2 circumvents physiological limitations on glutamate catabolism, preserves the amine nitrogen of glutamate for aspartate synthesis, and reduces production of toxic ammonia.

Across cultured cell lines, the majority of the carbons in aspartate and other TCA cycle intermediates are supplied by glutamine (73, 86). As a result, most cultured cells are exquisitely dependent upon glutamine to support anaplerosis. However, the extent to which cells depend on glutaminolysis for anaplerosis has been shown to depend on multiple factors, including glucose *versus* glutamine availability, cellular capacity to deal with ammonia toxicity, and overall cellular demand for glutamine, which also serves as a nitrogen source in biosynthetic pathways (78, 87). Anaplerotic substrate preference is likely also governed by cellular redox demands given that GDH-mediated production of αKG also produces NAD(P)H, which can be used for oxidative stress management and other biological processes (73, 78).

#### Pyruvate carboxylase

Pyruvate, derived from glucose, lactate, and amino acid sources, can function as a major anaplerotic source of OAA through the activity of the mitochondrial enzyme pyruvate carboxylase (PC) (37). PC catalyzes the ATP-dependent carboxylation of pyruvate to generate OAA (88). PC-derived OAA can either contribute to the TCA cycle or serve as a substrate for phosphoenolpyruvate carboxykinase, which catalyzes the decarboxylation of OAA to phosphoenolpyruvic acid in the gluconeogenic pathway (37). Consequently, while PC is expressed in most tissues, it is particularly active in gluconeogenic tissues like the kidney and liver. Even in nongluconeogenic tissues, PC responds to physiologic nutrient shifts to maintain anaplerosis. PC activity is allosterically activated by abundant acetyl-CoA, making it a potent sensor of OAA insufficiency in the mitochondria (89). PC is also allosterically inhibited by both αKG and glutamate, thereby suppressing simultaneous engagement of both PC- and glutamine-mediated anaplerosis (90). When glutamine anaplerosis is disrupted, PC becomes a critical source of OAA. PC is essential for growth of succinate dehydrogenase (SDH)–mutant cells, which exhibit a truncated TCA cycle and thus require an alternative source of OAA to support TCA cycle function and sustain aspartate production for anabolic pathways (91, 92). Likewise, cells that are addicted to glutamine become dependent on PC for growth when glutamine metabolism is suppressed; conversely, cells with high basal PC activity are generally more resistant to interruption of glutamine metabolism (87). Thus, while glutamine anaplerosis efficiently allows cells both to capture reducing equivalents from substrate oxidation and maintain OAA production, PC provides a critical backup when glutamine is not available or when nutrient status is so high that the ATP-dependent circumventing of the oxidative TCA cycle mediated by PC may actually help cells cope with nutrient overload.

#### Other sources of anaplerosis

Succinyl-CoA and fumarate are additional anaplerotic entry points within the TCA cycle. Mitochondrial β-oxidation of fatty acids with an odd number of carbon atoms yields acetyl-CoA and, ultimately, the 3-carbon propionyl-CoA, which cannot be further oxidized through the β-oxidation pathway. Rather, propionyl-CoA is converted to the TCA cycle intermediate succinyl-CoA through a pathway involving propionyl-CoA carboxylase and methylmalonyl-CoA mutase (5). Propionyl-CoA is also a product of the catabolism of certain essential amino acids, notably methionine, isoleucine, and valine (93). In addition, propionyl-CoA can be generated directly from circulating propionate, which is produced alongside other short-chain fatty acids by the gut microbiome (94, 95). Supraphysiologic propionate administration increases TCA cycle metabolite pools in the liver and induces hepatic gluconeogenesis (96), but the degree to which cells can activate propionyl-CoA catabolism to sustain anaplerotic flux under physiologic conditions remains largely unknown. Suggestively, some cultured cells maintain large propionyl-CoA pools fueled by isoleucine catabolism, and it will be interesting for future work to investigate the significance of this flux for TCA cycle metabolism and cell proliferation (97). A similar alternative entry point for amino acids into the TCA cycle is direct conversion of phenylalanine and tyrosine to fumarate by fumarylacetoacetate hydratase (5). Following entry into the pathway and oxidation to OAA, these anaplerotic substrates can support continuous cycling of the TCA cycle. The signals that may control these fluxes—and whether usage of these substrates modulates other aspects of TCA cycle wiring—remains to be explored.

### Electron shuttles

The reducing equivalent NADH is produced in the mitochondria through the reactions of the TCA cycle but is also a byproduct of glycolysis in the cytosol. Notably, while NAD^+^ itself can be imported into mitochondria through SLC25A51–MCART1 (98, 99, 100), NADH is not efficiently transported. The impermeability of the inner mitochondrial membrane to reduced electron carriers necessitates pathways that mediate the transport of reducing equivalents from the cytoplasm into the mitochondrial matrix for oxidation in the ETC. These so-called “electron shuttles” transport electrons by linking the oxidation of cytosolic NADH with reduction of a substrate that can be imported into the mitochondria and subsequently reoxidized. Beyond supporting net NADH “import” into the mitochondria, these shuttles also facilitate the oxidation of cytosolic NADH, which is required to support continued glycolysis and the *de novo* synthesis of serine and lipids (101, 102, 103). Electron shuttles that directly intersect with the TCA cycle or the ETC are discussed below.

#### Glycerol 3-phosphate shuttle

**Figure 4:**
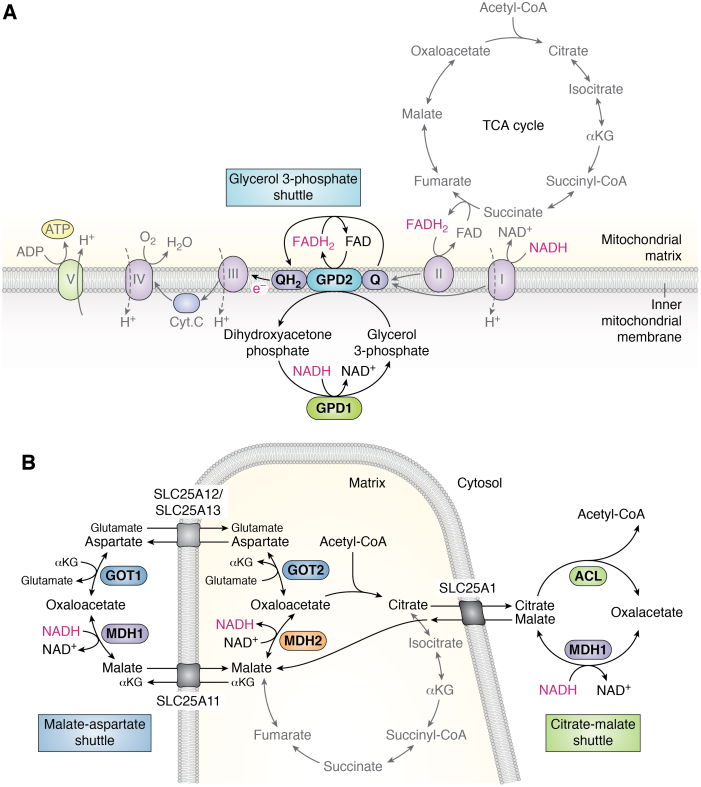
**Electron shuttles that intersect with the tricarboxylic acid (TCA) cycle or electron transport chain (ETC).***A*, in the glycerol 3-phosphate shuttle, the conversion of dihydroxyacetone phosphate into glycerol 3-phosphate by cytosolic glycerol 3-phosphate dehydrogenase 1 (GPD1) regenerates cytosolic NAD+ in the cytoplasm to support continued glycolysis. Glycerol 3-phosphate is subsequently converted back to dihydroxyacetone phosphate on the outer side of the inner mitochondrial membrane by mitochondrial glycerol 3-phosphate dehydrogenase 2 (GPD2), which is coupled with the conversion of FAD to FADH_2_. FADH_2_ donates electrons to ubiquinone (Q), reducing it to ubiquinol (QH_2_) that passes its electrons to complex III of the ETC. *B*, in the malate–aspartate shuttle (*left*), the TCA cycle intermediate oxaloacetate (OAA) and glutamate undergo transamination by glutamic-oxaloacetic transaminase 2 (GOT2), producing alpha-ketoglutarate (αKG) and aspartate. Mitochondrial aspartate is exported to the cytoplasm by the mitochondrial transporter proteins SLC25A12 or SLC25A13, and this efflux is concomitant with import of glutamate and a proton. Cytosolic aspartate is consumed by the cytosolic transaminase GOT1, converting αKG to glutamate and producing OAA. Cytosolic OAA is converted to malate by malate dehydrogenase 1 (MDH1), which is coupled with oxidation of NADH to NAD+, supporting glycolysis by regenerating NAD^+^ required for GAPDH. MDH1-generated malate is then reimported into mitochondria *via* the transporter SLC25A11, which is coupled with efflux of mitochondrial αKG. MDH2 converts malate to OAA, thereby reducing NAD^+^ to NADH and completing the cycle. In the citrate–malate shuttle (*right*), the citrate–malate antiporter SLC25A1 exports citrate to the cytosol, where it undergoes energy-dependent cleavage by ATP-citrate lyase (ACL), liberating acetyl-CoA and OAA. Conversion of OAA to malate by MDH1 supports NADH oxidation to NAD^+^, and this malate is imported into mitochondria in exchange for citrate by SLC25A1. In the mitochondria, malate is oxidized to OAA by MDH2, completing the shuttling of NADH into the mitochondrion.

The first electron shuttle to be discovered was the glycerol 3-phosphate shuttle, which was identified in insect flight muscle (104). While this shuttle does not involve TCA cycle intermediates, it does represent an electron input into the ETC. This pathway relies on two glycerol 3-phosphate dehydrogenase (GPD) enzymes, GPD1 and GPD2, located in the cytosol and mitochondrial membrane, respectively, to coordinate cytosolic NAD^+^ regeneration with electron donation to the ETC (Fig. 4*A*). The glycerol 3-phosphate shuttle is not considered the major route of NADH shuttling in most mammalian cells but has been shown to be particularly active in brown adipose tissue and may be implicated in thermoregulation (105, 106, 107). Moreover, activity of this pathway may become upregulated to compensate for disruption of other electron shuttles, for example, in the case of the developmental disorder caused by deficiency in the malate–aspartate shuttle enzyme malate dehydrogenase 1 (MDH1) (108).

#### Malate–aspartate shuttle

The malate–aspartate shuttle, or Borst cycle, was first proposed by Piet Borst in the early 1960s during his study of Ehrlich ascites tumor cells. He determined that it was improbable that any of the known electron shuttles of that time, including the glycerol 3-phosphate shuttle, were active in these cells and proposed the reactions of the malate–aspartate shuttle as a means of driving the oxidation of cytosolic NADH (104, 109). The malate–aspartate shuttle uses the complementary reactions of glutamic-oxaloacetic transaminase 1 and GOT2 transaminases paired with MDH1 and MDH2 to oxidize NADH in the cytosol and reduce NAD^+^ in the mitochondria (Fig. 4*B*). While theoretical at first, the reactions of the malate–aspartate shuttle provided an explanation for the earlier finding that GOT2 transaminase was highly active in the mitochondria and present in high concentrations that were proportional to those of TCA cycle enzymes (110). The components of the shuttle were verified *in vitro*, and the shuttle was quickly adopted as the predominant mechanism driving cytosolic NADH oxidation in heart and other mammalian tissues (107, 111). However, work by Krebs *et al.* (104, 112) indicated that the NAD^+^/NADH ratio is significantly higher in the cytosol than the mitochondria, making it unclear how electron shuttles could sustain the continuous oxidation of cytosolic NADH. Thus, the energetics underlying the shuttle were debated until the early 1970s when it was discovered that efflux of aspartate from the mitochondria was energy dependent and coupled to the import of a proton, allowing the shuttle to be powered by the proton motive force (113, 114). The coupling of aspartate efflux to proton import feature makes the malate–aspartate shuttle largely unidirectional toward glutamate import and aspartate efflux in most contexts (104). Upon ETC dysfunction, the cytosolic steps of the malate–aspartate shuttle can reverse to produce, rather than consume, cytosolic aspartate (42, 43).

Through shared metabolic intermediates and enzymes, the malate–aspartate shuttle and TCA cycle are functionally coupled (115). Moreover, given that aspartate efflux is dependent upon mitochondrial membrane potential, variability in TCA cycle and ETC function will both influence and be influenced by malate–aspartate shuttle activity (115). However, how TCA cycle flux and malate–aspartate shuttle activity are coordinated to support cross-compartment metabolism is poorly understood. Future work should aim to elucidate the mechanisms underlying how intermediates are partitioned between the TCA cycle and the malate–aspartate shuttle, including factors controlling the fate of OAA—whether continuing the TCA cycle or exiting as aspartate.

#### Citrate–malate shuttle

Prior to the discovery that the malate–aspartate shuttle was powered by the proton motive force, Piet Borst proposed the reactions of the citrate–malate shuttle as a potential alternative electron shuttle that could drive continuous NADH oxidation despite the cytosol's high NAD^+^/NADH ratio. While both shuttles rely on MDH1 and MDH2, the citrate–malate shuttle is coupled with an energy-expending reaction to favor cytosolic NADH oxidation (104, 116). Here, TCA cycle–derived citrate is transported by the citrate–malate antiporter SLC25A1 to the cytosol where energy (ATP) is required for cleavage by ACL, a reaction that liberates acetyl-CoA and OAA. OAA is then reduced to malate by MDH1, and malate is reimported into the mitochondria to complete the electron shuttle (Fig. 4*B*). Given that the mitochondrial membrane is impermeable to acetyl-CoA, it was appreciated early on that cleavage of cytosolic citrate by ACL provides a route for delivering acetyl-CoA into the cytosol and, thus, supporting fatty acid synthesis (117). However, experimental evidence that citrate cleavage also impacts NADH shuttling to the mitochondria came with the observation that hydroxycitrate, an inhibitor of ACL, significantly increases the lactate over pyruvate ratio, a proxy for the cytosolic NADH/NAD^+^ ratio, in rat livers perfused with ethanol (118). Citrate efflux from mitochondria has since been found to be a major process in some cancers, for example, cholesterol-rich rat hepatomas and and immune cells (38, 119, 120). More recently, it was demonstrated that the citrate–malate shuttle performs functions beyond that of an electron shuttle and, in fact, supports continuous citrate regeneration and TCA cycle intermediate homeostasis (119, 121). In cancer cells and embryonic stem cells (ESCs), the citrate–malate shuttle comprises a major alternative to the traditional TCA cycle and the degree to which cells engage the traditional TCA cycle as opposed to the citrate–malate shuttle, which may represent a “noncanonical TCA cycle,” is cell state dependent (121). Given that electron shuttles translocate the reducing equivalent (NADH) that both drives ETC activity and modulates flux through the TCA cycle, future work will likely continue to uncover functional links between these metabolic pathways and the TCA cycle.

## Flexibility of TCA cycle wiring

For many decades after its initial discovery, the TCA cycle was generally thought to operate as a fixed pathway in most cellular contexts. However, it has become increasingly appreciated that TCA cycle function is not “one size fits all,” and that components of the pathway are differentially and flexibly engaged in a context-specific fashion. For example, under conditions of hypoxia or impaired mitochondrial respiration, certain reactions of the TCA cycle reverse to support glutamine-dependent reductive carboxylation (92, 122, 123). During reductive carboxylation, glutamine-derived αKG is converted to isocitrate and then to citrate through reverse isocitrate dehydrogenase (IDH) and aconitase (ACO) activity, respectively (122, 123). Reductive carboxylation is favored under reducing conditions, and rescue of the mitochondrial NAD^+^/NADH ratio in cells with a defective ETC blunts engagement of this process (124, 125). As low mitochondrial NAD^+^/NADH inhibits production of acetyl-CoA by PDH, reductive carboxylation provides a critical alternative mechanism to generate citrate required to sustain cytosolic acetyl-CoA supply for *de novo* lipid synthesis even during reducing conditions. ACL cleavage of citrate derived from reductive carboxylation may also provide an alternative anaplerotic source of OAA, allowing cells to bypass a truncated or otherwise defective TCA cycle (123).

Beyond reductive carboxylation, other portions of the TCA cycle have also been shown to exhibit reversibility. Krebs found that GDH operates at close to equilibrium *in vitro*, and some evidence indicates that GDH may be reversible in some tissues *in vivo* (112, 126). For example, providing isotopically labeled ammonia to perfused livers or to rat portal veins results in the appearance of heavy labeled amino acids—reactions that would all depend on GDH as the initiating step for ammonia assimilation (127). Conditions of high ammonia availability may also favor reverse GDH activity as a means of ammonia detoxification and/or biomass accumulation. Indeed, breast cancer cells can engage in reductive amination, and GDH activity supports ammonia incorporation and tumor growth in mouse models of breast cancer (128).

Extremely reducing conditions can ultimately drive additional steps of the TCA cycle to operate in reverse. For example, ETC inhibition, which prevents cells from using molecular oxygen as a terminal electron acceptor, can force the SDH complex to operate in reverse, reducing fumarate to produce succinate (129). In this context, fumarate functions as an alternative electron acceptor, an adaptation that allows cells to sustain electron flow into the ETC despite oxygen limitation (129, 130, 131). By siphoning electrons onto fumarate, reverse SDH activity allows the ETC to continue to accept electrons from other oxidation reactions, such as that catalyzed by dihydroorotate dehydrogenase, which is critical for nucleotide biosynthesis (129). Even CS may reverse: CS reversibility, which occurs in prokaryotes under high CO_2_ conditions (see aforementioned), has been observed in mammalian cells in the context of SDH deficiency in which reductive glutamine metabolism produces citrate and, strikingly, citrate-derived OAA that can be used to sustain biosynthesis (132). Whether mitochondrial citrate cleavage by CS provides any specific advantages over cytoplasmic citrate cleavage by ACL, or whether reverse CS flux is secondary to biochemical conditions that disfavor citrate efflux, remains to be determined. More broadly, these reversible networks show that metabolic perturbations can ripple throughout cellular compartments and trigger pathway rewiring to ensure resilience in the face of stress.

## Nodes of TCA cycle control

Because the TCA cycle functions as a critical biosynthetic hub and is coupled with cellular energy production *via* OXPHOS, cells have evolved multiple nodes of TCA cycle regulation. In this section, we will outline three different mechanisms by which cells achieve tight control over TCA cycle activity.

### Allosteric regulation of TCA cycle enzyme activity

Metabolic flux through the TCA cycle is tightly coordinated by both negative and positive allosteric regulation of TCA cycle–associated enzymes. Three enzymes in particular—CS, IDH, and oxoglutarate dehydrogenase (OGDH)—catalyze rate-controlling steps in the TCA cycle and are considered regulatory enzymes within the pathway (41). All three enzymes undergo allosteric inhibition by high levels of NADH (41, 133). In the ETC, complex I and complex II oxidize NADH and FADH_2_, respectively, to provide the oxidized electron carriers required for continuous TCA cycle activity (134). High NADH thus serves as a key signal of ETC overloading to shutdown TCA cycle flux and prevent delivery of excess electrons to the ETC that can generate potentially dangerous reactive oxygen species (ROS). OXPHOS activity also signals to the TCA cycle at the level of ATP: high ATP/ADP ratios allosterically inhibit IDH, resulting in a deceleration of the cycle upon excess energy supply (135). OGDH undergoes substrate inhibition by succinyl-CoA, which also serves as an allosteric inhibitor of CS (134, 136). Apart from the three regulatory enzymes, SDH undergoes allosteric inhibition by OAA, slowing the cycle down upon insufficient acetyl-CoA availability (137). Altogether, allosteric regulation of core TCA cycle enzymes tunes the TCA cycle according to reactant and product availability, preventing excess nutrient catabolism in the absence of demand.

**Figure 5:**
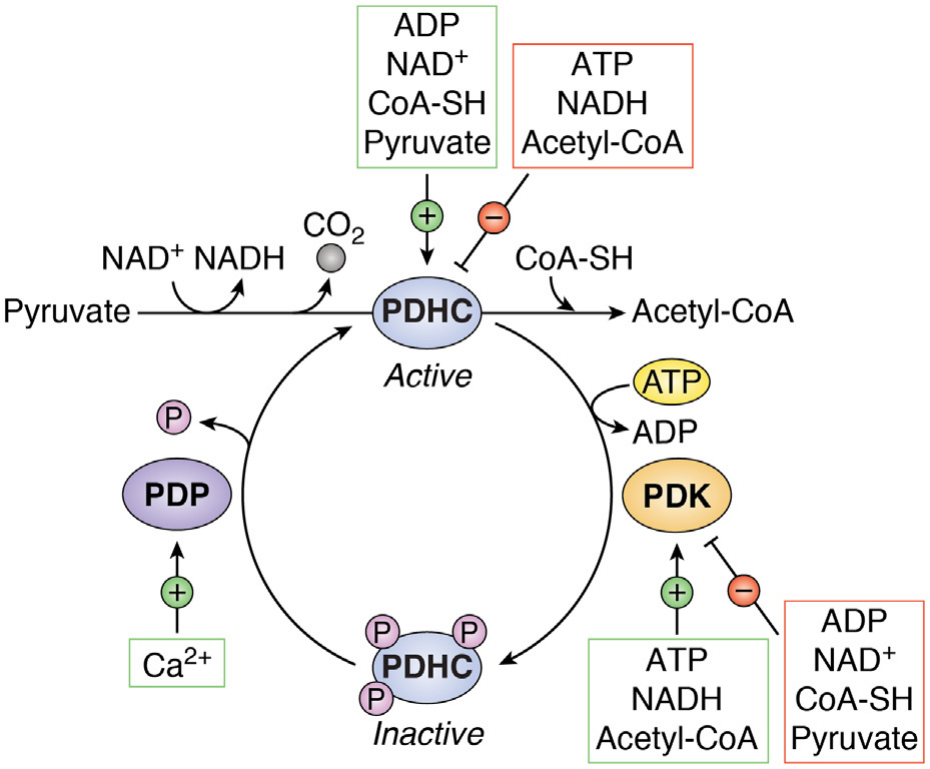
**Allosteric and covalent regulation of the pyruvate dehydrogenase (PDHC) complex.** The PDHC catalyzes the irreversible decarboxylation of pyruvate to acetyl-CoA, releasing CO_2_ and transferring electrons to NAD+ to form NADH. The PDHC utilizes multiple coenzymes, including coenzyme A (CoA-SH), during its multistep reaction. The complex is activated by increased levels of ADP, NAD^+^, CoA-SH, and its substrate pyruvate. Conversely, the PDHC is allosterically inhibited by high levels of ATP, NADH, and its product acetyl-CoA. Phosphorylation of any of three serine residues on the PDHC by pyruvate dehydrogenase kinases (PDKs) inactivates the complex. The PDKs are activated by high levels of ATP, NADH, and acetyl-CoA, reinforcing the shutdown of PDHC flux under these high-energy conditions. Reciprocally, the PDKs are inhibited by ADP, NAD^+^, CoA-SH, and pyruvate. The inhibitory phosphorylation of PDHC is reversible and can be removed by the pyruvate dehydrogenase phosphatases (PDPs), which are activated by mitochondrial calcium (Ca^2+^).

While not technically part of the TCA cycle, the PDHC generates the starting molecule of the cycle, acetyl-CoA, thereby functioning as a gatekeeper of TCA cycle activity. During PDHC catalysis, a carboxyl group is removed from pyruvate and released as CO_2_, and the remaining two-carbon molecule is oxidized, with NAD^+^ accepting the electrons to form NADH (Fig. 5). The PDHC is a multienzyme complex composed of multiple copies of three catalytic subunits: pyruvate dehydrogenase (E1), dihydrolipoamide acetyltransferase (E2), and dihydrolipoamide dehydrogenase (E3). These subunits carry out the sequential conversion of pyruvate into acetyl-CoA along with five different coenzymes, including coenzyme A (CoA-SH), the universal carrier for acyl groups. The complex is activated by its substrate, pyruvate, and inhibited by its product, acetyl-CoA. Like IDH, the PDHC is also allosterically inhibited by a high ATP/ADP ratio, allowing it to respond to cellular energy status. Importantly, like the three regulatory enzymes of the TCA cycle described previously, the PDHC is negatively regulated by high NADH levels. Inhibition of the complex by NADH links PDHC flux and, thus, acetyl-CoA production to ETC function and mitochondrial redox status (Fig. 5). Recent work has shown that enhancing pyruvate oxidation *via* the PDHC can result in a metabolic imbalance wherein NADH production outstrips NADH oxidation *via* the ETC, limiting the availability of NAD^+^ for key biosynthetic reactions such as aspartate biosynthesis (138). Increasing flux through OXPHOS by increasing ATP consumption reverses the growth inhibitory effects of PDHC activation (138). These results indicate that cell proliferation can be impaired when the demand for mitochondrial NAD^+^ exceeds that of ATP and may explain why highly proliferative cells often engage in aerobic glycolysis over pyruvate oxidation and OXPHOS, even in the presence of sufficient oxygen (138). By converting pyruvate to lactate in lieu of oxidizing it in the TCA cycle, cells can avoid excess production of NADH relative to ATP and the concomitant effect on proliferation that this produces.

### Covalent modification of TCA cycle–related enzymes

Beyond allosteric regulation, the PDHC is also regulated by covalent modification. The activity of the mammalian complex is regulated by the phosphorylation status of three serine residues on the pyruvate dehydrogenase E1 alpha subunit (139). Four pyruvate dehydrogenase kinases (PDKs; PDK1–4) catalyze phosphorylation of the serine residues, each with different site specificities (139). Phosphorylation of the complex is inhibitory but reversible *via* removal by pyruvate dehydrogenase phosphatases (PDPs; PDP1 and PDP2). While the PDHC is activated by NAD^+^, ADP, and pyruvate, the PDKs are conversely activated by NADH, ATP, and high acetyl-CoA levels (140) (Fig. 5). Collectively, the PDKs and PDPs provide cells with the ability to fine-tune PDHC activity through multiple signaling inputs. For example, low oxygen levels activate the hypoxia-inducible transcription factors (HIFs), which bind to hypoxia-response elements within a number of target genes including LDH-A (*LDHA*) and *PDK1*, leading to suppression of PDHC activity with a concomitant increase in aerobic glycolysis (141). To preferentially engage aerobic glycolysis, cancer cells frequently co-opt several pathways, including the HIF/hypoxia transcriptional program, to enhance PDK1 expression and achieve PDHC repression (142). Notably, the four PDKs exhibit different tissue and context specificities, which likely provides additional layers of PDHC control (143). Whether core TCA cycle enzymes are also controlled by phosphorylation remains to be shown, but there are some examples of TCA cycle enzymes like SDH undergoing alternative post-translational modification (144, 145, 146). Future work should aim to uncover other potential TCA cycle regulatory mechanisms exerted by covalent modification.

### Ion-mediated TCA cycle regulation

Calcium (Ca^2+^) functions as an intracellular messenger in an array of biological processes, including mitochondrial metabolism. Mitochondrial Ca^2+^ uptake primarily occurs *via* the mitochondrial calcium uniporter, a highly selective ion channel in the inner mitochondrial membrane. Mitochondrial calcium uniporter–mediated Ca^2+^ influx is driven by the membrane potential established by ETC activity (147). Once inside the mitochondria, Ca^2+^ ions directly activate IDH and OGDH enzymes. Ca^2+^ binding to IDH leads to a decrease of the *K*_*M*_ for isocitrate, and this effect is enhanced in the context of low ATP/ADP ratios (148). Similarly, Ca^2+^ binding to OGDH decreases the *K*_*M*_ for αKG. Intramitochondrial Ca^2+^ indirectly activates the PDHC by activating PDP1, leading to dephosphorylation and activation of the complex (148, 149). Thus, intramitochondrial Ca^2+^ regulates both carbon entry into and flux through the TCA cycle. Maintenance of Ca^2+^ homeostasis is critical for cell viability as mitochondrial Ca^2+^ overload favors opening of the permeability transition pore, leading to collapse of mitochondrial membrane potential and activation of cell death (147).

Iron also plays a significant role in modulating the TCA cycle by regulating both the activity and expression of ACO. Both the mitochondrial and cytosolic isoenzymes of ACO—ACO2 and ACO1, respectively—contain an iron–sulfur (4Fe–4S) cluster, and catalytic activity requires substrate coordination to a specific iron atom within this cluster. This enzymatic requirement for iron makes ACO activity sensitive to iron levels within cells (150). Iron-responsive elements (IREs) are motifs found within genes related to iron metabolism. Upon iron depletion, iron regulatory proteins bind to IREs in the 5′ end of mRNA transcripts, typically resulting in reduced mRNA translation and expression (151). The 5′ end of ACO2 contains a conserved IRE, which is likely responsible for the reduction in ACO2 protein expression observed in mice maintained on a low iron diet (152). Intriguingly, the 4Fe–4S cluster present within ACO2 makes it exquisitely susceptible to inactivation by ROS, serving as another example of TCA cycle deceleration upon ETC dysfunction (150). Other metal ions play significant roles in TCA cycle enzyme activity: IDH, OGDH, and PDP all bind Mg^2+^, but as free Mg^2+^ concentrations are considerably higher than enzyme *K*_*M*_s, whether fluctuations in Mg^2+^ are sufficient to modulate TCA cycle flux remains to be determined (153, 154, 155, 156).

## TCA cycle metabolites dictate cell fate and function

As a central metabolic hub, the TCA cycle frequently undergoes significant remodeling during both normal and pathological changes in cell fate. This section will cover some examples of how both inputs and outputs of the TCA cycle are exploited to alter cell function and, in turn, cell fate.

### Metabolic control of gene expression and cell fate

**Figure 6:**
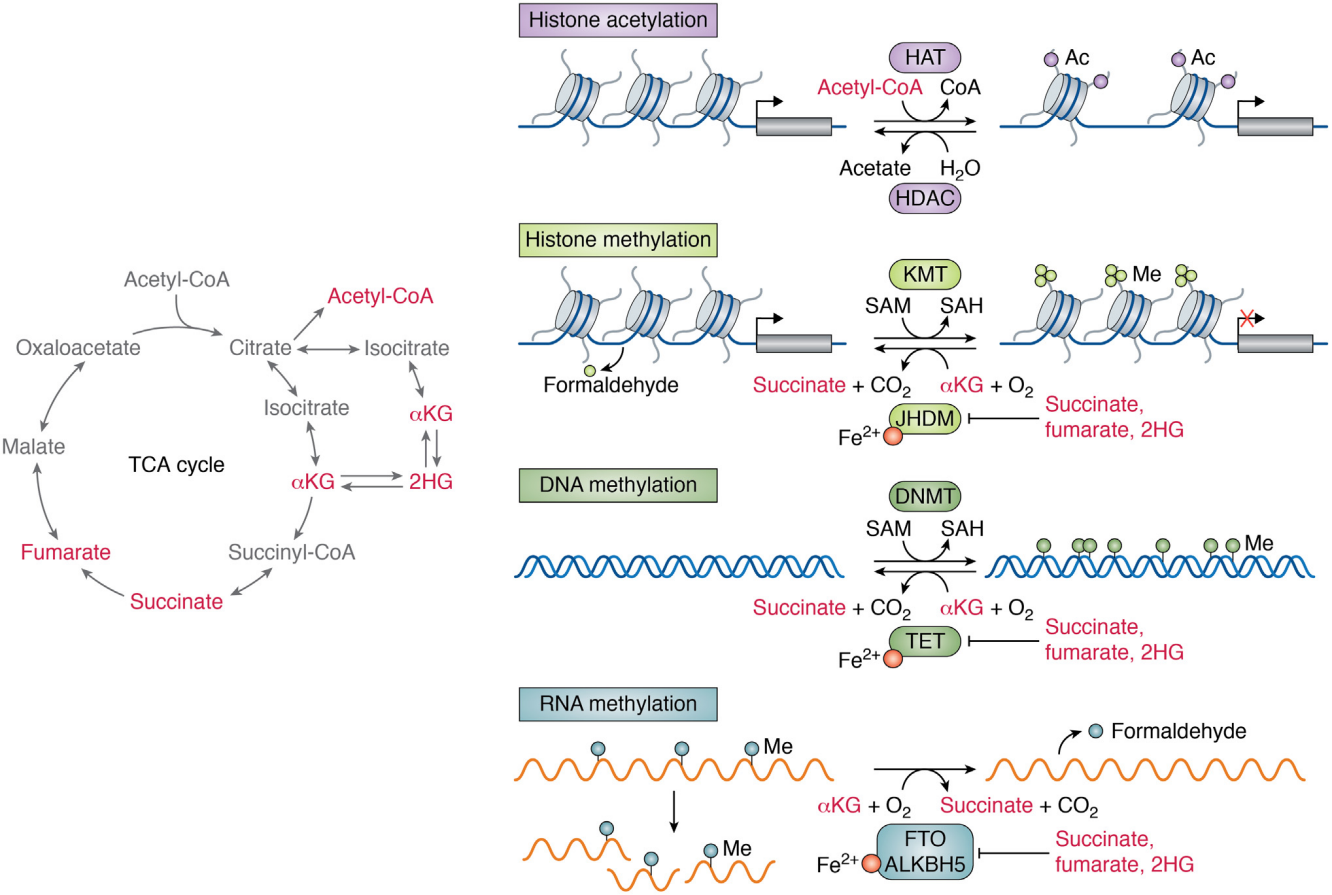
**Regulation of chromatin by tricarboxylic acid (TCA) cycle–associated metabolites.** Certain TCA cycle–derived metabolites (*left*) control the regulation of chromatin and, in turn, gene expression. Histone acetyltransferases (HAT) transfer acetyl groups from acetyl-CoA to histones, thereby altering chromatin accessibility. Histone deacetylases (HDACs) remove acetyl groups from histones and generate acetate as a product. Methylation on histones and DNA is deposited by histone lysine methyltransferases (KMTs) and DNA methyltransferases (DNMTs), respectively. DNMT and KMT add methyl groups by catalyzing the transfer of a methyl group from *S*-adenosyl methionine (SAM), producing *S*-adenosyl homocysteine (SAH). Alpha-ketoglutarate (αKG)-dependent dioxygenases regulate the demethylation of histones and nucleic acids. Jumonji C-domain–containing histone demethylases (JHDMs) and ten–eleven translocation (TET) DNA methylcytosine dioxygenases, which remove repressive histone marks and 5-methylcytosine, respectively, require αKG as an obligate cosubstrate and are competitively inhibited by succinate, fumarate, and 2-hydroxyglutarate (2HG). mRNA methylation is controlled by the RNA methyltransferases and αKG-dependent dioxygenases FTO and ALKBH5, which like all αKG-dependent dioxygenases are stimulated by αKG and repressed by succinate, fumarate, and 2HG. Histone and RNA demethylation produce formaldehyde as a byproduct.

A handful of TCA cycle intermediates are increasingly implicated in cell fate control. While transcription factors are the ultimate controllers of cell fate, their ability to act can be shaped by chemical modifications on DNA and histones that can modulate DNA accessibility and cofactor recruitment (157). Notably, steady-state levels of TCA cycle metabolites can affect the activity of chromatin-modifying enzymes, including αKG-dependent dioxygenases and histone acetyltransferases (Fig. 6). By co-opting the production and localization of select TCA cycle metabolites, cells can modulate the activity of chromatin-modifying enzymes and exert control over gene expression and cell fate (158, 159, 160).

Succinate, fumarate, and αKG are all implicated in regulation of αKG-dependent dioxygenases, a family of enzymes that includes Jumonji C-domain–containing histone demethylases and ten–eleven translocation (TET) DNA methylcytosine dioxygenases, which remove repressive histone marks and 5-methylcytosine, respectively (Fig. 6). These enzymes all require αKG, ferrous iron, and oxygen as cosubstrates and are inhibited by high levels of succinate and fumarate (161), thereby integrating multiple metabolic pathways in the control of gene expression programs and, ultimately, cell fate decisions.

#### Modulation of αKG production

Alterations in intracellular αKG levels have been shown to modulate cell fate in cancer and stem cells in a variety of model systems. The role of αKG in cell fate is most clearly demonstrated by studies that manipulate nutrient availability to modulate intracellular αKG pools. Depriving *APC*-mutant intestinal organoids of glutamine depletes intracellular αKG levels and promotes stem-like features and adenocarcinoma formation *in vivo* (162). αKG supplementation rescues these effects by driving DNA and histone demethylation, which facilitates upregulation of differentiation-associated genes and blunts tumor growth *in vivo* (162). Endogenous metabolic networks may determine αKG availability: the branched-chain amino acid transaminase 1 (BCAT1) enzyme, which transaminates αKG to initiate catabolism of valine, leucine, and isoleucine, constrains αKG pools in acute myelogenous leukemia. Accordingly, high BCAT1 activity leads to a hypermethylated chromatin landscape that blunts normal myeloid differentiation; reciprocally, blocking BCAT1 triggers αKG accumulation and induces myeloid differentiation (163).

Intracellular αKG pools may also be controlled by oncogenic mutations. In cell lines derived from mouse models of pancreatic ductal adenocarcinoma driven by mutant Kras and reversible silencing of the tumor suppressor p53, restoring p53 function rewires TCA cycle metabolism and drives accumulation of αKG relative to succinate (164). An increased αKG/succinate ratio in this system, whether driven by p53 reactivation or suppression of OGDH, enhances activity of αKG-dependent dioxygenases like the TETs and drives reacquisition of premalignant gene expression patterns associated with tumor differentiation (164). Together, these studies provide examples of how αKG accumulation—whether enabled by changes in TCA cycle wiring or upstream pathways—can function in a tumor suppressive capacity by stimulating chromatin remodeling that drives a more differentiated and less aggressive cancer phenotype. However, the effect of αKG on cell fate is likely context specific as interventions that increase αKG levels in mouse ESCs favor self-renewal over differentiation (165, 166). How αKG levels are set in cells—in particular, whether changes in mitochondrial TCA cycle wiring can result in alterations in nucleocytosolic αKG pools—and what determines cellular response to changes in αKG, remains to be determined.

#### Oncometabolite accumulation

Germline and somatic mutations in genes encoding TCA cycle enzymes are directly implicated in αKG-dependent dioxygenase activity. Mutations that impair fumarate hydratase (FH) and SDH activity are associated with pathologic accumulation of substrates fumarate and succinate, respectively. Oncogenic mutations in genes encoding IDH1/2 result in neomorphic enzyme activity that favors reduction of αKG to d-2-hydroxyglutarate (D-2HG) (167, 168). Succinate, fumarate, and 2HG are all considered “oncometabolites” because they are linked to development of certain human cancers and because they affect cancer-relevant processes by virtue of their ability to act as competitive inhibitors of αKG-dependent dioxygenases (169) (Fig. 6). IDH1/2 mutations have been identified in a range of tumor types, including solid tumors (*e.g.*, gliomas and chondrosarcomas) and blood cancers like acute myelogenous leukemia (169, 170, 171). By blocking the enzymatic activity of histone and DNA demethylases, d-2HG can function to lock cells in a hypermethylated state that blocks differentiation and reinforces a malignant and stem cell–like phenotype (30, 172, 173, 174). The enantiomer of d-2HG, l-2HG, can be produced in the absence of mutant IDH1/2 *via* the promiscuous enzymatic activity of MDH1, MDH2, or LDHA (169). This promiscuous enzymatic activity is amplified during hypoxia (175, 176) and under acidic pH (177, 178), raising the possibility that 2HG accumulation may play a regulatory role under normal physiological conditions.

Like 2HG accumulation, increased levels of succinate and fumarate can inhibit Jumonji C-domain–containing histone demethylases and TET enzymes, driving a widespread hypermethylation phenotype that disrupts normal cell differentiation (179, 180). Consistently, heterozygous germline mutations in genes encoding the TCA cycle enzymes involved in the breakdown of these metabolites—SDH and FH—have been shown to facilitate the pathogenesis of certain rare cancers (*e.g.*, paragangliomas) and inherited cancer-predisposition syndromes (181, 182, 183). In addition to affecting the activity of chromatin-associated αKG-dependent dioxygenases, succinate and fumarate accumulation can also inhibit αKG-dependent prolyl hydroxylases, leading to stabilization of HIF-1 and concomitant activation of the HIF/hypoxia transcriptional program (184, 185). As a result of this “pseudohypoxic” state, SDH- and FH-mutant tumors exhibit enhanced glycolytic metabolism and high ROS production (186). These tumors provide examples of how tumors can exploit TCA cycle metabolism to promote epigenetic and transcriptional remodeling that facilitates tumorigenesis.

#### Acetyl-CoA and histone acetylation

The TCA cycle provides another critical input to chromatin regulation in the form of acetyl-CoA. As discussed previously, several substrates feed into the TCA cycle at the level of acetyl-CoA, which condenses with OAA to form citrate. Acetyl-CoA itself cannot transit across the mitochondrial membrane. However, citrate export to the cytosol and subsequent cleavage by ACL provides a major source of cytosolic acetyl-CoA that is the obligate substrate for both *de novo* lipid synthesis and protein acetylation reactions (39, 187, 188). Acetylation of histone tails alters chromatin dynamics and generally increases accessibility to transcription factors, leading to activation of gene transcription (189) (Fig. 6). Thus, regulation of cytosolic acetyl-CoA generation can modulate transcriptional outputs by altering global histone acetylation (187). The PI3K–AKT signaling pathway mediates both the generation and cytosolic export of glucose-derived citrate, whereas AKT-mediated phosphorylation of ACL enhances its catalytic activity (190). Accordingly, oncogenic activation of the PI3K–AKT signaling axis increases acetyl-CoA levels and promotes elevated histone acetylation both *in vitro* and in tumors (188). These findings suggest that activation of signaling pathways stimulates transcriptional remodeling in part by altering levels of TCA cycle metabolites.

### TCA cycle metabolites in paracrine signaling

Beyond their intracellular roles, TCA cycle metabolites can also function as messengers facilitating interorgan crosstalk. To date, succinate represents the most established example of a TCA cycle intermediate that serves as a paracrine signal. During exercise, skeletal muscle responds to paracrine factors to undergo remodeling and tissue adaptation. Notably, both mouse and human muscle cells selectively release succinate during physical activity (191). This release occurs in a pH-dependent manner *via* monocarboxylate transporter 1 and is facilitated by transient protonation of succinate upon muscle cell acidification (191). Circulating succinate binds its receptor SUCNR1 in nonmyofibrillar cells in muscle tissue to initiate transcriptional programs that support tissue remodeling in response to exercise (191). In this system, two metabolic shifts—namely exercise-mediated acidification of muscle cells and succinate accumulation—coordinate to initiate a paracrine signaling cascade during physical activity. Succinate may also affect tissue function by directly controlling intracellular processes. For example, intracellular succinate accumulates rapidly upon cold temperature–mediated activation of thermogenesis in brown adipose tissue—in part because brown adipocytes begin sequestering circulating succinate more effectively (192). Succinate pools are directly oxidized by SDH, resulting in ROS production that stimulates uncoupling protein 1 activity, which in turn uncouples the mitochondrial proton gradient from respiration, thereby producing heat (192). Future work should continue to elucidate how succinate and other intracellular TCA cycle metabolites are converted to paracrine messengers that signal to the local environment and/or the rest of the organism.

## TCA cycle heterogeneity

### TCA cycle heterogeneity in cancer

Beyond inherent flexibility in reactions of the TCA cycle, different cell states and contexts also display variability in overall TCA cycle behavior, including preferred substrates for oxidation. Most mammalian cells growing in culture rely on both glucose- and glutamine-derived carbon to fuel the TCA cycle. However, isotope tracing studies in both lung and glioblastoma tumors suggest that glutamine is a relatively minor source of TCA cycle carbon in some cancer cells growing *in vivo*, indicating that environmental context can drive fuel preferences (193, 194). Recent tracing studies have also revealed that TCA cycle metabolism *in vivo* is fueled by substrates beyond glucose and glutamine. As described previously, the contribution of circulating lactate to TCA cycle intermediates exceeds that of glucose in most tissues and tumors (46, 47). Some cancer types, including liver and glioblastomas, readily take up acetate, which can be converted to acetyl-CoA by ACSS and undergo oxidation in the TCA cycle (70, 195).

Beyond being driven by environmental differences between *in vitro versus in vivo* growth, TCA cycle substrate preferences are also determined by cancer tissue type and driver genetic mutations. For example, liver tumors with aberrant overexpression of *MYC* display enhanced glutamine oxidation and sensitivity to therapies that target glutamine catabolism relative to liver tumors driven by overexpression of *MET* (196). Clear cell renal carcinomas exhibit a tumor type–specific metabolic profile: these tumors do not oxidize glucose-derived carbon in the TCA cycle and instead preferentially engage in aerobic glycolysis (197). Altogether, advances in *in vivo* metabolic tracing are uncovering a wide array of metabolic strategies employed by tumors, and future work should continue to elucidate how oncogenes, lineage, environment, and cell state collectively determine metabolic phenotypes and liabilities of cancer cells.

### TCA cycle heterogeneity in immune cells

The field of immunometabolism was ignited following the discovery that activated T-cells significantly upregulate glucose uptake and glycolysis to support the energetic and biosynthetic demands of T-cell expansion (198). Since this seminal finding, it has become increasingly appreciated that the TCA cycle also undergoes significant remodeling during immune cell activation and that this remodeling supports distinct immune cell functions and fates. For example, activated macrophages increase expression of the enzyme aconitate decarboxylase 1 (previously known as IRG1), which produces itaconate from the decarboxylation of TCA cycle–derived aconitate, an intermediate in the conversion of citrate to isocitrate (199). Itaconate displays anti-inflammatory properties, in part because of its ability to activate the transcription factor Nrf2, which has antioxidant and anti-inflammatory activity (200). Activated macrophages have also been shown to accumulate high levels of succinate, which induces the proinflammatory cytokine interleukin 1β (201). In response to T-cell receptor stimulation, 2HG accumulates to millimolar levels in CD8+ T cells and enhances effector differentiation through a mechanism involving altered histone and DNA methylation (202). In natural killer cells, cytokine activation induces Srebp-dependent metabolic reprogramming that drives metabolism of glucose-derived citrate through the citrate–malate shuttle; accordingly, blocking the citrate–malate shuttle or Srebp activation inhibits natural killer cell effector function (119). These are just a few examples of the enormous potential of TCA cycle reprogramming to reinforce immune cell function and alter immune cell fate. More examples of TCA cycle–driven immunomodulation can be found in recent reviews (3, 134).

### TCA cycle heterogeneity in stem cells

Adult tissue stem cells maintain organ homeostasis by balancing self-renewal and differentiation into mature cell types. Intriguingly, this functional plasticity may be accompanied by notable metabolic plasticity. In multiple tissue contexts, adult stem cells have been found to primarily engage in aerobic glycolysis. Upon lineage commitment and differentiation, however, cells increase their oxidation of pyruvate in the TCA cycle and display enhanced OXPHOS (203, 204, 205). For example, intestinal stem cells (ISCs) express lower levels of MPC than their differentiated progeny, and blocking mitochondrial pyruvate entry *via* MPC inhibition drives increased ISC numbers and proliferative potential (206). Unlike ISCs, hair follicle stem cells (HFSCs) are not continuously proliferative and instead remain dormant unless induced to divide during a new hair cycle (207). LDHA expression and activity are high in HFSCs relative to other cell types in the epidermis, and promoting lactate production *via* MPC deletion boosts their activation (208). Conversely, deleting LDHA prevents HFSC activation and the initiation of a new hair cycle (208). Myogenic differentiation is also coupled with increased pyruvate oxidation and OXPHOS, and this metabolic shift is likely driven, in part, by the significant energy demands of skeletal muscle (121, 204, 209). Collectively, these studies illustrate that TCA cycle substrate preference and behavior can vary within the same lineage in adult stem cells.

Pluripotent stem cells are emerging as a powerful system in which to study metabolism during cell-state transitions. While pluripotency, or the ability to give rise to all embryonic germ layers, is a state that exists only transiently during early mammalian development, it can be modeled indefinitely *in vitro* using ESCs derived from the inner cell mass of preimplantation blastocysts (210). Mouse and human ESCs can be cultured under conditions that drive a spectrum of pluripotent states, ranging from those that closely mimic the preimplantation state (otherwise known as the “naïve” state) to those that resemble the postimplantation epiblast. Increasing work is identifying metabolic alterations that correlate with developmental stages in both mouse and human, including variability in preferred substrate for oxidation in the TCA cycle and changes in steady-state levels of TCA cycle metabolites. While most cultured cell lines require glutamine supplementation to support cell survival and proliferation, naïve mouse ESCs can grow in the absence of exogenous glutamine. The glutamine-independent phenotype exhibited by naïve mouse ESCs is coupled with a reduction in the contribution of glutamine-derived carbons to TCA cycle intermediates (165). Moreover, naïve mouse ESCs accumulate pools of αKG and acetyl-CoA, which may reinforce the pluripotent state by stimulating more open chromatin (165, 211, 212, 213, 214). TCA cycle activity also varies with PSC identity in human ESCs, which display glutamine independence and increased mitochondrial respiration upon conversion to a more naïve state (215, 216). Pluripotent cell-state transitions can be accompanied by large-scale changes in TCA cycle configuration. For example, we recently showed that naïve ESCs primarily oxidize citrate in the traditional TCA cycle, and, accordingly, loss of ACO2 compromises acquisition of naïve pluripotency (121). Conversely, cells undergoing exit from the naïve pluripotent state shift toward cytosolic TCA cycle metabolism *via* the citrate–malate shuttle. Consequently, blocking the citrate–malate shuttle by disrupting ACL causes significant defects in TCA cycle metabolite homeostasis and prevents ESCs from exiting from the naïve pluripotent state (121). These findings reveal that variability in TCA cycle wiring may drive the metabolic heterogeneity observed across ESCs and other mammalian cell types.

## Conclusions

Although the core biochemical steps of the TCA cycle were elucidated over a half-century ago, research continues to yield new insight into the regulation and function of the TCA cycle in mammalian cells. Diverse cell culture model systems and *in vivo* isotope tracing approaches have combined to illuminate notable heterogeneity in TCA cycle substrate preference and wiring in both normal development and pathological conditions. How this diversity is achieved—and to what extent cells can transit in and out of different metabolic states—remain major open questions. Advances in functional screening alongside approaches to study metabolism *in vivo* with increasing resolution will reveal both cell type–specific metabolic preferences and dependencies that can be targeted to manipulate cell growth or homeostasis. More broadly, given the centrality of the TCA cycle to all pathways in cell metabolism, it will be important to continue to investigate how metabolic networks are coordinated across cellular compartments and how changes in TCA cycle activity signal to modulate key cellular functions such as differentiation. A deepened understanding of how specific signaling and environmental cues shape TCA cycle behavior will strengthen future endeavors to modulate the growth and survival of specific cell types for therapeutic purposes.

## Conflict of interest

P. K. A. and L. W. S. F. are authors on patent applications that cover links between cellular metabolism and cell fate control.
